# On *t*-derivations of PMS-algebras

**DOI:** 10.12688/f1000research.159711.1

**Published:** 2025-01-13

**Authors:** Nibret Melese Kassahun, Berhanu Assaye Alaba, Yohannes Gedamu Wondifraw, Zelalem Teshome Wale

**Affiliations:** 1 Mathematics, Bahir Dar University College of Science, Bahir Dar, Amhara, 6000, Ethiopia; 2 Mathematics, Debre Markos University College of Natural and Computational Science, Debre Markos, Amhara, None, Ethiopia; 3 Mathematics, Addis Ababa University College of Natural Sciences, Addis Ababa, Addis Ababa, 1000, Ethiopia

**Keywords:** PMS-algebras, derivations, t-derivations, semigroup

## Abstract

**Background:**

PMS algebras are a type of algebraic structure that has been studied extensively in recent years. They are a generalization of several other algebraic structures, such as Boolean algebras and MV-algebras.

**Methods:**

In this paper, we introduce the concept of t-derivations on PMS algebras. T-derivations are a type of mapping between PMS algebras that satisfies certain properties. We then study the properties of t-derivations and regular t-derivations on PMS algebras.

**Results:**

We characterize further properties of t-derivations in the context of PMS algebras. We also investigate a novel result of t-derivations on the G-part of a PMS-algebra. Finally, we prove that the set of all t-derivations on a PMS-algebra forms a semigroup.

**Conclusions:**

This paper provides a comprehensive study of t-derivations on PMS algebras. We have established several new results and characterized the properties of t-derivations in detail. Our results contribute to the further understanding of PMS algebras and their associated structures.

## 1. Introduction

Iseki ^3^ introduced BCK-algebras, which are a type of abstract algebra, in his 1978 work. Additionally, Iseki ^4^ established another class of abstract algebras called BCI- algebras, which are an extension of BCK-algebras, in his research work. Furthermore, Iseki ^4^ studied and explored the algebraic properties of BCI-algebras. Also, Tamalibrasi and Megalai ^8^ introduced and studied TM-algebras based on propositional calculus. Moreover, Mostafa et al. ^9^ studied the concepts of KUS-algebras, KUS-ideals, and KUS-subalgebras, and their relationships were investigated. Sithar and Nagalakshmi ^11^ continued studying. PMS-algebras, which encompass the notions of BCK/BCI/TM/KUS algebras. They also discussed the ideals of PMS-algebras and studied their properties.

Derivations are essential areas of study in algebraic structure theory, originating from invariant theory and Galois theory. Derivations were introduced in rings and near rings by many researchers. Based on derivations in rings and near-rings theory, Jun and Xin ^7^ expanded the notion of derivations to BCI-algebras. After derivations were intro- duced in various algebraic structures, derivations were extended to new concepts called t-derivations. As a result, t-derivations are studied and characterized in various algebraic structures. Muhiuddin and Al-Roqi ^10^ introduced the notion of t-derivations on BCI- algebras and characterized their interesting results based on the context of BCI-algebras in their 2012 work. In 2013, Ganeshkumar and Chandramouleeswaran ^2^ introduced the idea of t-derivations on TM-algebras, and they explored their basic properties. Jana et al. ^5^ established the notion of t-derivations on subtraction algebras with their essential properties in their 2017 work. Furthermore, in 2020, t-derivations were introduced on lattices and explored their properties based on lattice structures by Javed et al. ^6^ Re- cently, in 2021, Siswanti et al. ^12^ t-derivations were studied on BP-algebras, and their detailed properties were examined. Additionally, Ganesan and Kandaraj ^1^ t-derivations on BH-algebras were introduced in their 2021 research work. In this paper, we introduce t-derivations on PMS-algebras and investigate their nice properties based on the structure of PMS-algebras. We also define regular derivations on PMS-algebras and characterize their properties. Finally, we show that the set of all t-derivations on PMS-algebras is a semigroup.

## 2. Preliminaries

In this section, we will address the concepts and basic properties of PMS-algebras that we need for the main results in the next sections. The following definitions and properties are taken from Sithar and Nagalakshmi. ^11^

Definition 2.1. A PMS-algebra is an algebra (X; ⋆, 0) of type (2, 0) with a constant “0” and a binary operation “⋆” satisfying the following axioms:

(1) 0 ⋆ a = a;
(2) (b ⋆ a) ⋆ (c ⋆ a) = c ⋆ b for all a, b, c ∈ X.

In X, we define a binary relation “≤” by: a ≤ b if and only if a ⋆ b = 0.

Proposition 2.2. *In a* PMS *-algebra* (X; ⋆, 0) *the following properties hold for all* a, b, c ∈ X.

(1) a ⋆ a = 0.
(2) (b ⋆ a) ⋆ a = b.
(3) a ⋆ (b ⋆ a) = b ⋆ 0.
(4) (b ⋆ a) ⋆ c = (c ⋆ a) ⋆ b.
(5) (a ⋆ b) ⋆ 0 = b ⋆ a = (0 ⋆ b) ⋆ (0 ⋆ a).

Definition 2.3. Let S be a nonempty subset of a PMS-algebra (X; ⋆, 0). Then S is called a PMS-subalgebra of X if a ∗ b ∈ S for all a, b ∈ S.

Definition 2.4. Let X be a PMS-algebra. The set G (X) = {a ∈ X: a⋆ 0 = a} is called the G-part of X.

Definition 2.5. Let X be a PMS-algebra. The set B (X) = {a ∈ X: a⋆ 0 = 0} is called the p-radical of X.

## 3. t-derivations

Remark 3.1. Let (X; ⋆, 0) be a PMS-algebra.

(1) If b ⋆ a = c ⋆ a, then b = c;
(2) If a ⋆ b = a ⋆ c, then b = c for all a, b, c ∈ X.

Proof.
Let a, b, c ∈ X.

(1) Assume that b ⋆ a = c ⋆ a
Now b = 0 ⋆ b = (a ⋆ a) ⋆ b = (b ⋆ a) ⋆ a = (c ⋆ a) ⋆ a = (a ⋆ a) ⋆ c = 0 ⋆ c = c.
(2) Suppose that a ⋆ b = a ⋆ c
Now b ⋆ a = b ⋆ ((a ⋆ b) ⋆ b) = b ⋆ ((a ⋆ c) ⋆ b) = (a ⋆ c) ⋆ 0 = (0 ⋆ c) ⋆ a = c ⋆ a. Hence by (1), we get b = c. ■

Definition 3.2. A PMS-algebra X is said to be associative if (x ⋆ y) ⋆ z = x ⋆ (y ⋆ z) for all x, y, z ∈ X.

Example 3.3. Let X be the PMS-algebra with the following Cayley table.

**Table:** 

⋆	0	1	2	3
0	0	1	2	3
1	1	0	3	2
2	2	3	0	1
3	3	2	1	0

Then (X; ⋆, 0) is an associative PMS-algebra.

Definition 3.4. Let X be a PMS-algebra. Then, for t ∈ X, define a self-map d *_t_*: X→ X by d *_t_* (x) = t ⋆ x for all x ∈ X.

Definition 3.5. Let X be a PMS-algebra. Then, for t ∈ X, a self-map d *_t_*: X→ X is said to be a (left, right)-t-derivation of X, denoted by, (l, r)-t-derivation if d *_t_* (x ⋆ y) = (d *_t_* (x) ⋆ y) ∧ (x ⋆ d *_t_* (y)) for all x, y ∈ X.

Remark 3.6. Let d *_t_* be a (l, r)-t-derivation on a PMS-algebra X. Then d *_t_* (x ⋆ y) = d *_t_* (x) ⋆ y for all x, y ∈ X.

Example 3.7. Let X be an associative PMS-algebra with the following Cayley Table.

**Table:** 

⋆	0	1	2
0	0	1	2
1	1	0	1
2	2	1	0

Define, when t = 0, d *_t_* (x) = x, ∀x ∈ X. when t = 1, d *_t_* (0) = 1, d *_t_* (1) = 0, d *_t_* (2) = 1. when t = 2, d *_t_* (0) = 2, d *_t_* (1) = 1, d *_t_* (2) = 0. Therefore, for each t ∈ X, d *_t_* is a (l, r)-t-derivation on X.

Definition 3.8. Let X be a PMS-algebra. Then, for t ∈ X, a self-map d *_t_*: X→ X is said to be a (l, r)-t-derivation of X, denoted by, (r, l)-t-derivation if d *_t_* (x ⋆ y) = (x ⋆ d *_t_* (y)) ∧ (d *_t_* (x) ⋆ y) for all x, y ∈ X.

Remark 3.9. Let d *_t_* be a (r, l)-t-derivation on a PMS-algebra X. Then d *_t_* (x ⋆ y) = x ⋆ d *_t_* (y) for all x, y ∈ X.

Example 3.10. Let X be a non-associative PMS-algebra with the following Cayley Table.

**Table:** 

⋆	0	1	2
0	0	1	2
1	2	0	1
2	1	2	0

Define, when t = 0, d *_t_* (x) = x, ∀x ∈ X. when t = 1, d *_t_* (0) = 2, d *_t_* (1) = 0, d *_t_* (2) = 1.

when t = 2, d *_t_* (0) = 1, d *_t_* (1) = 2, d *_t_* (2) = 0.

Therefore, for each t ∈ X, d *_t_* is a (r, l)-t-derivation on X.

Proposition 3.11. *Let* d *_t_ be a self-map on a PMS-algebra* X *. Then* d *_t_ is a* (r, l) *-*t *-derivation on* X.

Proof.
Let d *_t_*: X→ X defined by d *_t_* (x) = t ⋆ x. Now for x, y ∈ X,

$$\begin{array}{l} d_{t}\left(x⋆y\right)=t⋆\left(x⋆y\right)\\ \;=\left(\left(t⋆y\right)⋆y\right)⋆\left(x⋆y\right)\\ \;=x⋆\left(t⋆y\right)\\ \;=x⋆d_{t}(t⋆y) \end{array}$$

Hence d *_t_* is a (r, l)-t-derivation on X. ■

Definition 3.12. Let X be a PMS-algebra. Then, for t ∈ X, a self-map d *_t_*: X→ X is called the t-derivation on X if d *_t_* is both a (l, r)-t-derivation and a (r, l)-t-derivation on X.

Example 3.13. Consider the PMS-algebra X in Example 3.3. Define the mapping d *_t_* as follows:

When t = 0, d *_t_* (x) = x, ∀x ∈ X.

When t = 1, d *_t_* (0) = 1, d *_t_* (1) = 0, d *_t_* (2) = 3, d *_t_* (3) = 2.

When t = 2, d *_t_* (0) = 2, d *_t_* (1) = 3, d *_t_* (2) = 0, d *_t_* (3) = 1.

When t = 3, d *_t_* (0) = 3, d *_t_* (1) = 2, d *_t_* (2) = 1, d *_t_* (3) = 0. Therefore, for each t ∈ X, d *_t_* is a t-derivation of X.

Proposition 3.14. *Let* d *_t_ be a self-map on an associative* PMS *-algebra* X *. Then* d *_t_ is a* (l, r) *-*t *-derivation on* X.

Proof.
Let X be an associative PMS-algebra and d *_t_* be a self-map on X.

$$\begin{array}{l} d_{t}\left(x⋆y\right)=t⋆\left(x⋆y\right)\\ \;=\left(t⋆x\right)⋆y\\ \;=d_{t}\left(x\right)⋆y \end{array}$$

Hence d *_t_* is (r, l)-t-derivation on X.

By combining Proposition 3.11 and Proposition 3.14, we get the following theorem. ■

Theorem 3.15. *Let* X *be an associative* PMS *-algebra. For t* ∈ X *, a self-map* d *_t_ is a* t *-derivation on* X.

Definition 3.16. A self-map d *_t_* on a PMS-algebra X is said to be t-regular if d *_t_* (0) = 0.

Example 3.17. In Example 3.13, d *_t_* is a regular t-derivation on X where t = 0. However, for t = 1, t = 2, or t = 3, d *_t_* is not a regular t-derivation on X.

Proposition 3.18. *Let* X *be the* p *-radical of a* PMS *-algebra and* d *_t_ be a self-map on* X *. Then* d *_t_ is* t *-regular.*

Proof.
Let X be a p-radical PMS-algebra.

Let x ∈ X. Then x ⋆ 0 = 0. Now d *_t_* (0) = t ⋆ 0 = 0. Hence d *_t_* is t-regular. ■

Theorem 3.19. *Let* d *_t_ be a self-map on a* PMS *-algebra* X *. Then*

(1) *If* d *_t_ is a* (l, r) *-*t *-derivation on* X *, then* d *_t_* (x) = x ∧ d *_t_* (x) ∀x ∈ X *if and only if* d *_t_ is* t *-regular.*
(2) *If* d *_t_ is a* (r, l) *-*t *-derivation on* X *. If* d *_t_ is* t *-regular on* X *, then* d *_t_* (x) = d *_t_* (x) ∧ x ∀x ∈ X.

Proof.
(1) Let d *_t_* is a (l, r)-t-derivation on X. Suppose that d *_t_* (x) = x ∧ d *_t_* (x).

d *_t_* (0) = 0 ∧ d *_t_* (0) = (0 ⋆ d *_t_* (0)) ⋆ d *_t_* (0) = (d *_t_* (0) ⋆ d *_t_* (0)) ⋆ 0 = 0 ⋆ 0 = 0.

d *_t_* is regualr on X.

Conversely, assume that d *_t_* is regular.

Now d *_t_* (x) = d *_t_* (0 ⋆ x) = (d *_t_* (0) ⋆ x) ∧ (0 ⋆ d *_t_* (x)) = (0 ⋆ x) ∧ d *_t_* (x) = x ∧ d *_t_* (x). Hence d *_t_* (x) = x ∧ d *_t_* (x)

(2) Let d *_t_* is a (r, l)-t-derivation on X.

Suppose d *_t_* (0) = 0.

Now d *_t_* (x) = d *_t_* (0 ⋆ x) = (0 ⋆ d *_t_* (x) ∧ (d *_t_* (0) ⋆ x) = d *_t_* (x) ∧ (0 ⋆ x) = d *_t_* (x) ∧ x ■

Theorem 3.20. *Let* d *_t_ be a* (r, l) *-*t *-derivation on a* PMS *-algebra. Then the following holds true.*

(1) d *_t_* (0) = x ⋆ d *_t_* (x), ∀x ∈ X.
(2) d *_t_ is one-to-one.*
(3) *If* d *_t_ is* t *-regular, then it is the identity map.*
(4) *If there is an element* x ∈ X *such that* d *_t_* (x) = x *, then* d *_t_ is the identity map.*
(5) *If* x ≤ y *, then* d *_t_* (x) ≤ d *_t_* (y) *for all* x, y ∈ X.

Proof.
Let d *_t_* be a (r, l)-t-derivation on a PMS-algebra.

(1) Let x ∈ X. Then d *_t_* (0) = t ⋆ 0 = t ⋆ (x ⋆ x) = x ⋆ (t ⋆ x) = x ⋆ d *_t_* (x).
(2) Let x, y ∈ X such that d *_t_* (x) = d *_t_* (y). Then t ⋆ x = t ⋆ y. Now
$$\begin{array}{l} x⋆t=x⋆\left(\left(t⋆x\right)⋆x\right)\\ \;=x⋆\left(\left(t⋆y\right)⋆x\right)\\ \;=\left(0⋆x\right)⋆\left(\left(t⋆y\right)⋆x\right)\\ \;=\left(t⋆y\right)⋆0\\ \;=\left(0⋆y\right)⋆t\\ \;=y⋆t. \end{array}$$
Hence by right cancellation, x = y. Therefore d *_t_* is an identity map.
(3) Let d *_t_* be a t-regualr derivation on X.
By (1), we have 0 = d *_t_* (0) = x ⋆ d *_t_* (x) implies that x ⋆ x = x ⋆ d *_t_* (x) Hence by left cancellation, d *_t_* (x) = x.
(4) Let d *_t_* (x) = x for some x ∈ X.
Now 0 = x ⋆ x = x ⋆ d *_t_* (x) = d *_t_* (x ⋆ x) = d *_t_* (0) implies that d *_t_* is regular. Hence by (3) d *_t_* is an identity map.
(5) Let x ≤ y. Then x ⋆ y = 0.
Now d *_t_* (x)⋆d *_t_* (y) = (t⋆x)⋆(t⋆y) = ((t⋆y)⋆x)⋆t = ((x⋆y)⋆t)⋆t = (t⋆t)⋆(x⋆y) = 0 ⋆ 0 = 0.
Hence d *_t_* (x) ≤ d *_t_* (y). ■

Theorem 3.21. *Let* X *be a* PMS *-algebra and* d *_t_ be a* t *-derivation on* X *. If* y ≤ x *and* d *_t_* (x ⋆ y) = d *_t_* (x) ⋆ d *_t_* (y) *for all* x, y ∈ X *,t hen* d *_t_* (x) = d *_t_* (y).

Proof.
Let x ≤ y and d *_t_* (x ⋆ y) = d *_t_* (x) ⋆ d *_t_* (y).

Now for x ∈ X, d *_t_* (x) = d *_t_* (0⋆x) = d *_t_* ((y⋆x)⋆x) = d *_t_* (y⋆x)⋆d *_t_* (x) = (d *_t_* (y)⋆d *_t_* (x))⋆d *_t_* (x) = (d *_t_* (x) ⋆ d *_t_* (x)) ⋆ d *_t_* (y) = 0 ⋆ d *_t_* (y) = d *_t_* (y).

Hence d *_t_* (x) = d *_t_* (y).■

Theorem 3.22. *Let* d *_t_ be a* t *-regular* (l, r) *-*t *-derivation on a* PMS *-algebra* X *. Then the following hold.*

(1) d *_t_* (x) = x.
(2) d *_t_* (x) ⋆ y = x ⋆ d *_t_* (y) *for all* x, y ∈ X.
(3) d *_t_* (x ⋆ y) = d *_t_* (x) ⋆ y = d *_t_* (x) ⋆ d *_t_* (y) = x ⋆ y.
(4) Ker(d *_t_*) = {x ∈ X: d *_t_* (x) = 0} *is a subalgebra of* X *.*

Proof.
Let d *_t_* be a t-regular (l, r)-t-derivation on a PMS-algebra X.

(1) Let x ∈ X. Then d *_t_* (x) = d *_t_* (0 ⋆ x) = d *_t_* (0) ⋆ x = 0 ⋆ x = x.
(2) Let x, y ∈ X. Then d *_t_* (x) ⋆ y = x ⋆ y = x ⋆ d *_t_* (y).
(3) Let x, y ∈ X. Then d *_t_* (x ⋆ y) = d *_t_* (x) ⋆ y = d *_t_* (x) ⋆ d *_t_* (y) = x ⋆ y.
(4) Since d *_t_* is regular, d *_t_* (0) = 0. Hence 0 ∈ Ker(d *_t_*) Thus ker(d *_t_*) is nonempty.
Let x, y ∈ Ker(d *_t_*). Then d *_t_* (x) = 0, d *_t_* (Y) = 0. Now d *_t_* (x ⋆ y) = x ⋆ y = d *_t_* (x) ⋆ d *_t_* (y) = 0 ⋆ 0 = 0. Hence x ⋆ y ∈ Ker(d *_t_*).
Therefore Ker(d *_t_*) is a subalgebra of X. ■

Theorem 3.23. *Let* X *be a* PMS *-algebra. Then* Ker(d *_t_*) = {0} *if and only if* d *_t_ is* t *-regular.*

Proof.
Suppose Ker(d *_t_*) = {0}. Then d *_t_* (x) = 0 ∀x ∈ X. In particular, d *_t_* (0) = 0. Hence d *_t_* is regular.

Conversely, assume d *_t_* is regular. Let x ∈ Ker(d *_t_*). Then d _t_(x) = 0. Now

$$0=d_{t}\left(0\right)=d_{t}\left(x⋆x\right)=d_{t}\left(x\right)⋆x=0⋆x=x.$$

Thus x = 0 implies that Ker(d *_t_*) = {0} ■

Definition 3.24. Let X be a PMS-algebra and d *_t_* $d_{t}^{\prime }$ be self-maps on X. Then define a mapping d *_t_* ◦ $d_{t}^{\prime }$ : X → X by (d *_t_* ◦ $d_{t}^{\prime }$ )(x) = d *_t_* ( $d_{t}^{\prime }$ (x)) for all x ∈ X.

Theorem 3.25. *Let* X *be a* PMS *-algebra and* d *_t_* $d_{t}^{\prime }$ *be a* (l, r) *-*t *-derivation on* X *. Then* (d *_t_* ◦ $d_{t}^{\prime }$ ) *is a* (l, r) *-*t *-derivation on* X.

Proof.
Let X be a PMS-algebra and d *_t_* $d_{t}^{\prime }$ be a (l, r)-t-derivation on X.

For x, y ∈ X, we have

$$\begin{array}{l} \left(d_{t}◦d_{t}^{\prime }\right)\left(x⋆y\right)=d_{t}\left(d_{t}^{\prime }\left(x⋆y\right)\right)\\ \;=d_{t}\left(d_{t}^{\prime }\left(x\right)⋆y\right)\\ \;=d_{t}\left(d_{t}^{\prime }\left(x\right)\right)⋆y\\ \;=\left(d_{t}\;◦\;d_{t}^{\prime }\right)\left(x\right)⋆y \end{array}$$

Hence (d *_t_* ◦ $d_{t}^{\prime }$ ) is a (l, r)- t-derivation of X. ■

Theorem 3.26. *Let* X *be a PMS algebra and* d *_t_* d ^'^ *_t_ be a* (r, l) *-* t *-derivation of* X *. Then* (d *_t_* ◦ $d_{t}^{\prime }$ ) *is a* (r, l) *-*t *-derivation on* X.

Proof.
Let X be a PMS-algebra and d *_t_* $d_{t}^{\prime }$ be a (r, l)-t-derivation on X.

For x, y ∈ X, we have

$$\begin{array}{l} \left(d_{t}\;◦\;d_{t}^{\prime }\right)\left(x⋆y\right)=d_{t}\left(d_{t}^{\prime }\left(x⋆y\right)\right)\\ \;=d_{t}\left(x⋆d_{t}^{\prime }\left(y\right)\right)\\ \;=x⋆d_{t}\left(d_{t}^{\prime }\left(y\right)\right)\\ \;=x⋆\left(d_{t}\;◦\;d_{t}^{\prime }\right)\left(y\right) \end{array}$$

Hence (d *_t_* ◦ $d_{t}^{\prime }$ ) is a (r, l)-t-derivation of X.

Combining **Theorem** 3.25 and **Theorem** 3.26, we get the following theorem. ■

Theorem 3.27. *Let* X *be a* PMS *-algebra and* d *_t_* $d_{t}^{\prime }$ *be* t *-derivation of* X *. Then* (d *_t_* ◦ $d_{t}^{\prime }$ ) *is a* t *-derivation on* X.

Theorem 3.28. *Let* X *be a* PMS *-algebra. Let* d *_t_ be a* (r, l) *-*t *-derivation on* X *and* $d_{t}^{\prime }$ *be a* (l, r)-t- *derivation on* X. *Then* d _t_ ◦ $d_{t}^{\prime }$ = $d_{t}^{\prime }$ ◦ d _t_.

Proof.
Let d *_t_* be a (r, l)-t-derivation on X and $d_{t}^{\prime }$ be a (l, r)-t-derivation on X. Now

$$\begin{array}{l} \left(d_{t}\;◦\;d_{t}^{\prime }\right)\left(x\right)=\left(d_{t}\;◦\;d_{t}^{\prime }\right)\left(0⋆x\right)\\ \;=d_{t}\left(d_{t}^{\prime }\left(0⋆x\right)\right)\\ \;=d_{t}\left(d_{t}^{\prime }\left(0\right)⋆x\right)\\ \;=d_{t}^{\prime }\left(0\right)⋆d_{t}\left(x\right) \end{array}$$ (3.1)

$$\begin{array}{l} \left(d_{t}^{\prime }\;◦\;d_{t}\right)\left(x\right)=\left(d_{t}^{\prime }\;◦\;d_{t}\right)\left(0⋆x\right)\\ \;=d_{t}^{\prime }\left(d_{t}\left(0⋆x\right)\right)\\ \;=d_{t}^{\prime }\left(0⋆d_{t}\left(x\right)\right)\\ \;=d_{t}^{\prime }\left(0\right)⋆d_{t}\left(x\right) \end{array}$$ (3.2)

From (3.1) and (3.2) we have, (d *_t_* ◦ $d_{t}^{\prime }$ )(x) = ( $d_{t}^{\prime }$ ◦ d *_t_*)(x), ∀x ∈ X. Thus d *_t_* ◦ $d_{t}^{\prime }$ = $d_{t}^{\prime }$ ◦ d _t_. ■

Theorem 3.29. *Let* X *be a* PMS *-algebra and* d *_t_* $d_{t}^{\prime }$ *be two* t *-derivations on* X *, then* d *_t_* ◦ $d_{t}^{\prime }$ = $d_{t}^{\prime }$ ◦ d *_t_.*

Definition 3.30. Let X be a PMS-algebra and d *_t_* $d_{t}^{\prime }$ be self-maps on X. Then define d *_t_* ⋆ $d_{t}^{\prime }$ : X→ X by (d *_t_* ⋆ $d_{t}^{\prime }$ )(x) = d *_t_* (x) ⋆ $d_{t}^{\prime }$ (x) for all x ∈ X.

Theorem 3.31. *Let* X *be a* PMS *-algebra and* d *_t_* $d_{t}^{\prime }$ *be* t *-derivations on* X *. Then* d *_t_* ⋆ $d_{t}^{\prime }$ = $d_{t}^{\prime }$ ⋆ d *_t_.*

Definition 3.32. Let LDer *_t_* (X) be the set of all (l, r)-t-derivations on a PMS-algebra X. Then define a binary operation “∧” on LDer *_t_* (X) by (d *_t_* ∧ $d_{t}^{\prime }$ )(x) = d *_t_* (x) ∧ $d_{t}^{\prime }$ (x), ∀x ∈ X, where d *_t_* $d_{t}^{\prime }$ ∈ LDer *_t_* (X).

Lemma 3.33. *If* d *_t_ and* d ^'^ *_t_ are* (l, r) *-*t *-derivations on* X *. Then* (d *_t_* ∧ $d_{t}^{\prime }$ ) *is a* (l, r) *-*t *-derivation on* X.

Proof.
$$\begin{array}{l} \left(d_{t}\wedge d_{t}^{\prime }\right)\left(x⋆y\right)=d_{t}\left(x⋆y\right)\wedge d_{t}^{\prime }\left(x⋆Y\right)\\ \;=\left(d_{t}\left(x\right)⋆y\right)\wedge \left(d_{t}^{\prime }\left(x\right)⋆y\right)\\ \;=\left(\left(d_{t}\left(x\right)⋆y\right)⋆\left(d_{t}^{\prime }\left(x\right)⋆y\right)\right)⋆\left(d_{t}^{\prime }\left(x\right)⋆y\right)\\ \;=\left(\left(d_{t}^{\prime }\left(x\right)⋆y\right)⋆\left(d_{t}^{\prime }\left(x\right)⋆y\right)\right)⋆\left(d_{t}\left(x\right)⋆y\right)\\ \;=0⋆\left(d_{t}\left(x\right)⋆y\right)\\ \;=d_{t}\left(x\right)⋆y \end{array}$$ (3.3)

$$\begin{array}{l} \left(d_{t}\wedge d_{t}^{\prime }\right)\left(x\right)⋆y=\left(d_{t}\left(x\right)\wedge d_{t}^{\prime }\left(x\right)\right)⋆y\\ \;=\left(\left(d_{t}\left(x\right)⋆d_{t}^{\prime }\left(x\right)\right)⋆d_{t}^{\prime }\left(x\right)\right)⋆y\\ \;=\left(\left(d_{t}^{\prime }\left(x\right)⋆d_{t}^{\prime }\left(x\right)\right)⋆d_{t}\left(x\right)\right)⋆y\\ \;=\left(0⋆d_{t}\left(x\right)\right)⋆y\\ \;=d_{t}\left(x\right)⋆y \end{array}$$ (3.4)

From (3.3) and (3.4), we get (d *_t_* ∧ $d_{t}^{\prime }$ )(x ⋆ y) = (d *_t_* ∧ $d_{t}^{\prime }$ )(x) ⋆ y. ■

Lemma 3.34. *The binary operation* ∧ *defined on* L *_t_* Der(X) *is associative.*

Proof.
Let d *_t_* $d_{t}^{\prime }$ $d_{t}^{\prime \prime }$ ∈ LDer *_t_* (X). Now for x ∈ X,

$$\begin{array}{l} \left(\left(d_{t}\wedge d_{t}^{\prime }\right)\wedge d_{t}\prime \prime \right)\left(x\right)=\left(d_{t}\left(x\right)\wedge d_{t}^{\prime }\right)\left(x\right)\wedge d_{t}^{\prime \prime }\left(x\right)\\ \;\left(\left(d_{t}\left(x\right)⋆d_{t}^{\prime }\left(x\right)\right)⋆d_{t}^{\prime }\left(x\right)\right)\wedge d_{t}^{\prime \prime }\left(x\right)\\ \;\left(\left(d_{t}^{\prime }\left(x\right)⋆d_{t}^{\prime }\left(x\right)\right)⋆d_{t}\left(x\right)\right)\wedge d_{t}^{\prime \prime }\left(x\right)\\ \;=\left(0⋆d_{t}\left(x\right)\right)\wedge d_{t}^{\prime \prime }\left(x\right)\\ \;=d_{t}\left(x\right)\wedge d_{t}^{\prime \prime }\left(x\right)\\ \;=\left(d_{t}\left(x\right)⋆d_{t}^{\prime \prime }\left(x\right)\right)⋆d_{t}^{\prime \prime }\left(x\right)\\ \;=\left(d_{t}^{\prime \prime }\left(x\right)⋆d_{t}^{\prime \prime }\left(x\right)\right)⋆d_{t}\left(x\right)\\ \;=0⋆d_{t}\left(x\right)\\ \;=d_{t}\left(x\right) \end{array}$$ (3.5)

$$\begin{array}{l} \left(d_{t}\wedge \left(d_{t}^{\prime }\wedge d_{t}^{\prime \prime }\right)\right)\left(x\right)=d_{t}\left(x\right)\wedge \left(d_{t}^{\prime }\wedge d_{t}^{\prime \prime }\right)\left(x\right)\\ \;=d_{t}\left(x\right)\wedge \left(\left(d_{t}^{\prime }\left(x\right)⋆d\prime \prime_{t}\left(x\right)\right)⋆d\prime \prime_{t}\left(x\right)\right)\\ \;=d_{t}\left(x\right)\wedge \left(\left(d{\prime \prime }_{t}\left(x\right)⋆d{\prime \prime }_{t}\left(x\right)\right)⋆d_{t}^{\prime }\left(x\right)\right)\\ \;=d_{t}\left(x\right)\wedge \left(0⋆d_{t}^{\prime }\left(x\right)\right)\\ \;=d_{t}\left(x\right)\wedge d_{t}^{\prime }\left(x\right)\\ \;=\left(d_{t}\left(x\right)⋆d_{t}^{\prime }\left(x\right)\right)⋆d_{t}^{\prime }\left(x\right)\\ \;=\left(d_{t}^{\prime }\left(x\right)⋆d_{t}^{\prime }\left(x\right)\right)⋆d_{t}\left(x\right)\\ \;=0⋆d_{t}\left(x\right)\\ \;=d_{t}\left(x\right) \end{array}$$ (3.6)

From (3.5) and (3.6), we have ((d *_t_* ∧ $d_{t}^{\prime }$ ) ∧ $d_{t}^{\prime \prime }$ )(x) = (d *_t_* ∧ ( $d_{t}^{\prime }$ ∧ $d_{t}^{\prime \prime }$ ))(x), ∀x ∈ X.

Thus “∧” is associative. ■

Theorem 3.35. L *_t_* Der(X) *is a semigroup under the binary operation* ∧ *defined by* (d *_t_* ∧ $d_{t}^{\prime }$ )(x) = d *_t_* (x) ∧ $d_{t}^{\prime }$ (x) *for all* x ∈ X *, and* d *_t_* $d_{t}^{\prime }$ ∈ L *_t_* Der(X).

Definition 3.36. Let R *_t_* Der(X) be the set of all (r, l)-t-derivations on a PMS-algebra X. Then define a binary operation ∧ on R *_t_* Der(X) by (d *_t_* ∧ $d_{t}^{\prime }$ )(x) = d *_t_* (x) ∧ $d_{t}^{\prime }$ (x), ∀x ∈ X, where d *_t_* $d_{t}^{\prime }$ ∈ R *_t_* Der(X).

Lemma 3.37. *If* d *_t_ and* d ^'^ *_t_ are* (r, l) *-*t *-derivations on* X *. Then* (d *_t_* ∧ $d_{t}^{\prime }$ ) *is also a* (r, l) *-*t *-derivation on* X.

Proof.
Let d *_t_* $d_{t}^{\prime }$ ∈ RDer *_t_* (X). For

$$\begin{array}{l} x\in X,\;(d_{t}\wedge d_{t}^{\prime })\left(x⋆y\right)=d_{t}\left(x⋆y\right)\wedge d_{t}^{\prime }\left(x⋆y\right)\\ \;=\left(x⋆d_{t}\left(y\right)\right)\wedge \left(x⋆d_{t}^{\prime }\left(y\right)\right)\\ \;=\left(\left(x⋆d_{t}\left(y\right)\right)⋆\left(x⋆d_{t}^{\prime }\left(y\right)\right)\right)⋆\left(x⋆d_{t}^{\prime }\left(y\right)\right)\\ \;=\left(\left(x⋆d_{t}^{\prime }\left(y\right)\right)⋆\left(x⋆d_{t}^{\prime }\left(y\right)\right)\right)⋆\left(x⋆d_{t}\left(y\right)\right)\\ \;=x⋆d_{t}\left(y\right) \end{array}$$ (3.7)

$$\begin{array}{l} x⋆\left(d_{t}\wedge d_{t}^{\prime }\right)\left(y\right)=x⋆\left(d_{t}\left(y\right)\wedge d_{t}^{\prime }\left(Y\right)\right)\\ \;=x⋆\left(\left(d_{t}\left(y\right)⋆d_{t}^{\prime }\left(y\right)\right)⋆d_{t}^{\prime }\left(y\right)\right)\\ \;=x\;⋆((d_{t}^{\prime }\left(y\right)\;⋆d_{t}^{\prime }\left(y\right))\;⋆d_{t}(y))\\ \;=x\;⋆(0\;⋆d_{t}(y))\\ \;=x⋆d_{t}\left(y\right) \end{array}$$ (3.8)

From (3.7) and (3.8), we have, (d *_t_* ∧ $d_{t}^{\prime }$ )(x ⋆ y) = x ⋆ (d *_t_* ∧ $d_{t}^{\prime }$ )(y). ■

Lemma 3.38. *The binary operation “*∧ *” defined on* RDer *_t_* (X) *is associative.*

Proof.
Let d *_t_* $d_{t}^{\prime }$ $d_{t}^{\prime \prime }$ ∈ RDer *_t_* (X). Now for, x ∈ X.

$$\begin{array}{l} \left(\left(d_{t}\wedge d_{t}^{\prime }\right)\wedge d_{t}^{\prime \prime }\right)\left(x\right)=\left(\left(d_{t}\wedge d_{t}^{\prime }\right)\wedge d_{t}^{\prime \prime }\right)\left(0⋆x\right)\\ \;=(d_{t}\wedge d_{t}^{\prime })\left(0⋆x\right)\wedge d_{t}^{\prime \prime }\left(0⋆x\right)\\ \;=\left(d_{t}\left(0⋆x\right)\wedge d_{t}^{\prime }\left(0⋆x\right)\right)\wedge d_{t}^{\prime \prime }\left(0⋆x\right)\\ \;=\left(\left(0⋆d_{t}\left(x\right)\right)\wedge \left(0⋆d_{t}^{\prime }\left(x\right)\right)\right)\wedge \left(0⋆d{\prime \prime }_{t}\left(x\right)\right)\\ \;=\left(d_{t}\left(x\right)\wedge d_{t}^{\prime }\left(x\right)\right)\wedge d_{t}^{\prime \prime }\left(X\right)\\ \;=(d_{t}(x)⋆d_{t}^{\prime }(x))\;⋆d_{t}\prime \prime (x)\\ \;=(d_{t}\prime \prime (x)\;⋆d_{t}^{\prime }(x))\;⋆d_{t}(x)\\ \;=d_{t}\left(x\right) \end{array}$$ (3.9)

And

$$\begin{array}{l} \left(d_{t}\wedge (d_{t}^{\prime }\wedge d_{t}^{\prime \prime }\right))\left(x\right)=\left(d_{t}\wedge (d_{t}^{\prime }\wedge d_{t}^{\prime \prime }\right))\left(0\;⋆x\right)\\ \;=d_{t}(0⋆x)\wedge (d_{t}^{\prime }\wedge d_{t}^{\prime \prime })(0⋆x)\\ \;=(0\;⋆d_{t}(x))\;\wedge ((d_{t}^{\prime }(0⋆x)\;\wedge \;d_{t}^{\prime \prime }(0⋆x))\\ \;=d_{t}(x)\wedge ((0\;⋆d_{t}\prime (x))\;\wedge (0⋆d_{t}^{\prime \prime }(x)))\\ \;=d_{t}(x)\;\wedge (d_{t}^{\prime }(x)\wedge d_{t}\prime \prime (x))\\ \;=d_{t}(x)\;\wedge d_{t}^{\prime }(x)\\ \;=d_{t}(x) \end{array}$$ (3.10)

From (3.9) and (3.10), we have ((d *_t_* ∧ $d_{t}^{\prime }$ ) ∧ $d_{t}^{\prime \prime }$ )(x) = (d *_t_* ∧ ( $d_{t}^{\prime }$ ∧ $d_{t}^{\prime \prime }$ ))(x), ∀x ∈ X. Thus the binary operation “∧” is associative on RDer *_t_* (X). ■

Theorem 3.39. RDer *_t_* (X) *is a semigroup under the binary operation “*∧ *” defined by* (d *_t_* ∧ $d_{t}^{\prime }$ )(x) = d *_t_* (x) ∧ $d_{t}^{\prime }$ (x) *for all* x ∈ X *, where* d *_t_* $d_{t}^{\prime }$ ∈ R *_t_* Der(X).

Combining Theorem (3.35) and Theorem (3.39), we get the following theorem.

Theorem 3.40. *If* Der *_t_* (X) *denotes the set of all* t *-derivations on* X *. Then it is a semigroup under the binary operation* “∧” *defined by* (d *_t_* ∧ $d_{t}^{\prime }$ )(x) = d *_t_* (x) ∧ $d_{t}^{\prime }$ (x) *for all* x ∈ X *, where* d *_t_* $d_{t}^{\prime }$ ∈ Der *_t_* (X).

## Conclusions

The study of propositional logic-based algebraic structures, such as PMS-algebras, is crucial for understanding derivation concepts. Derivations are applied to coding theory. The concept of t-derivations in PMS-algebras is explored, and it is proven that the set of all t-derivations forms a semigroup. This research can be applied to other algebras like BF- algebras, UP-algebras, and KUS-algebras, providing a foundation for further research on derivations.

## Ethics and consent

Ethical approval and consent were not required.

## Data Availability

No data are associated with this article.
