# Supplementary material for: On t-derivations of PMS-algebras
Source: F1000Res. 2025 Jan 13;14:70. [Version 1] doi: 10.12688/f1000research.159711.1 (PMC12206288; doi:10.12688/f1000research.159711.1)
Supplement: Peer review history

# Reviewer report: Reviewer response for version 1

**DOI:** 10.5256/f1000research.175478.r375060

**Published:** 2025-06-28

**Authors:** Hemavathi P, Dr. Vinod Kumar R

**Affiliations:** 1 Mathematics, Rajalakshmi Engineering College, Thandalam/Chennai, Tamil Nadu, India; 2 Saveetha Institute of Medical and Technical Sciences (SIMATS), Thandalam, India

The manuscript presents a rigorous and comprehensive study of t-derivations in PMS-algebras, an emerging algebraic framework that generalizes several known systems such as BCK-, BCI-, TM-, and KUS-algebras. The paper introduces and explores both left-right and right-left t-derivations, regular t-derivations, and their algebraic behavior within PMS-algebras, culminating in the significant result that the set of all t-derivations forms a semigroup.

**Strengths**

- The topic is mathematically **relevant and original**, expanding derivation concepts to a broader class of algebraic systems.
- The **definitions are precise**, and the theorems are stated clearly with logically consistent proofs.
- The paper is well-structured, gradually developing the theory with **examples and counterexamples** to support understanding.
- The establishment of **semigroup properties** for derivation mappings is a valuable algebraic contribution.

**Concerns and Suggestions**

1. While the paper is mathematically sound, some **phrases are awkwardly constructed**, which may affect reader comprehension. A **language refinement** or minor copy-editing is recommended to improve clarity (e.g., consistent use of “self-map”, “identity map”, etc.).
2. veral notations such as dt, d’t, ∧, and various operation compositions (◦, ⋆) are heavily reused. A **notation table** at the beginning or end would be helpful for reference.
3. The introduction could be strengthened by briefly discussing **potential applications or motivations** for studying derivations in PMS-algebras, especially in logic, coding theory, or information systems, as hinted in the conclusion.

**Minor Comments**

- Fix typographical issues (e.g., “regualr” → “regular”; inconsistent capitalizations in definitions and remarks).
- The references are appropriate but could benefit from **standardized formatting** for better consistency (some include first names; others don’t).

Consider revising the **abstract** to be more informative currently, it repeats “we study” and “we prove” without summarizing the key findings in a concise, technical tone.

**Recommendation:**

**Minor Revision**

 The manuscript provides a well-structured and significant contribution to algebraic logic and derivation theory. A minor revision focusing on language, presentation, and notational clarity will enhance the paper’s accessibility and impact.

Is the work clearly and accurately presented and does it cite the current literature?

Yes

If applicable, is the statistical analysis and its interpretation appropriate?

Not applicable

Are all the source data underlying the results available to ensure full reproducibility?

No source data required

Is the study design appropriate and is the work technically sound?

Yes

Are the conclusions drawn adequately supported by the results?

Yes

Are sufficient details of methods and analysis provided to allow replication by others?

Yes

Reviewer Expertise:

Fuzzy algebra, Fuzzy Decision Making, Neutrosophic Sets

We confirm that we have read this submission and believe that we have an appropriate level of expertise to confirm that it is of an acceptable scientific standard.

# Reviewer report: Reviewer response for version 1

**DOI:** 10.5256/f1000research.175478.r375059

**Published:** 2025-05-26

**Authors:** Phani Yedlapalli

**Affiliations:** 1 Shri Vishnu Engineering College for Women, Vishnupur, India

The paper presents a novel study on *t-derivations* and *regular derivations* in PMS-algebras, introducing  concepts with mathematical rigor. The authors successfully explore key properties of these derivations, grounded in the structural framework of PMS-algebras. A notable result is that the set of all t-derivations forms a semigroup, which adds depth to the algebraic structure. The work is original, clearly written, and logically organized. The results have potential for further exploration and applications. Overall, this is a valuable and publishable contribution to algebraic research.

Is the work clearly and accurately presented and does it cite the current literature?

Yes

If applicable, is the statistical analysis and its interpretation appropriate?

Yes

Are all the source data underlying the results available to ensure full reproducibility?

Yes

Is the study design appropriate and is the work technically sound?

Yes

Are the conclusions drawn adequately supported by the results?

Yes

Are sufficient details of methods and analysis provided to allow replication by others?

Yes

Reviewer Expertise:

Algebras

I confirm that I have read this submission and believe that I have an appropriate level of expertise to confirm that it is of an acceptable scientific standard.
